# Supplementary material for: Analysis of Volatile and Non-Volatile Components of Dried Chili Pepper (Capsicum annuum L.)
Source: Foods. 2025 Feb 20;14(5):712. doi: 10.3390/foods14050712 (PMC11898792; doi:10.3390/foods14050712)
Supplement: Supplementary file 1 [file foods-14-00712-s001.zip › foods-3456206-Supplementary figures.pdf]

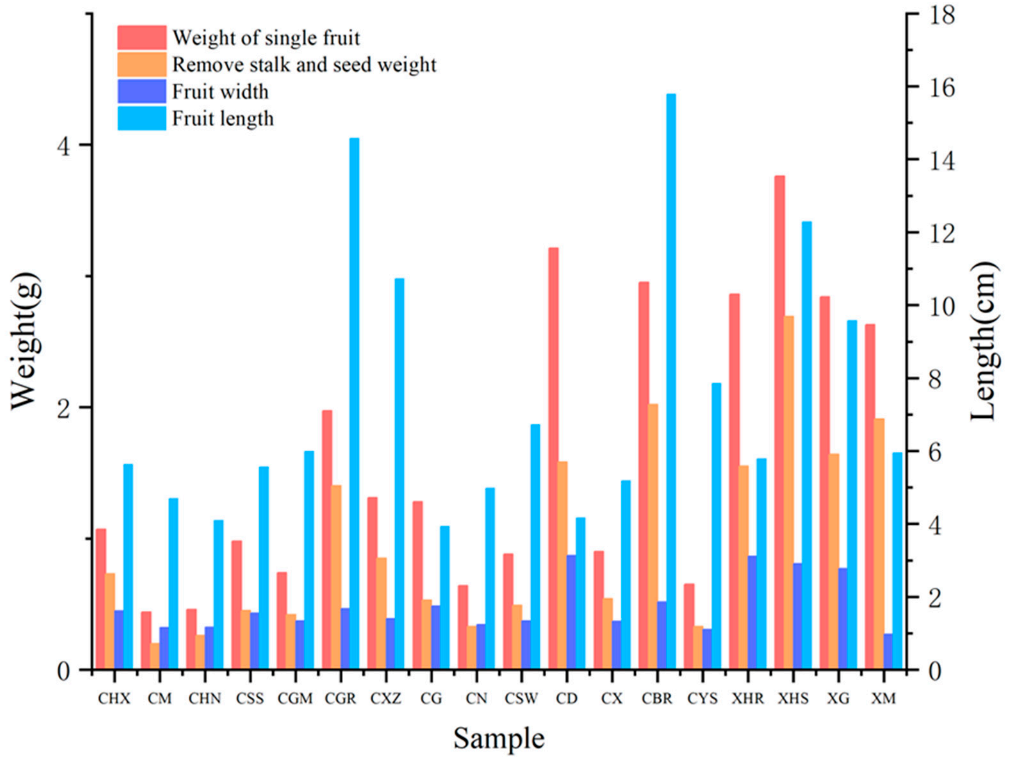

Figure S1. The total weight of different dried peppers, the weight of the stems and seeds, the length of the fruit, and the width of the fruit. (Weight of single fruit and Remove stalk and seed weight are expressed by g of the principal Y-axis, and Fruit length and Fruit width are expressed by cm of the secondary Y-axis)

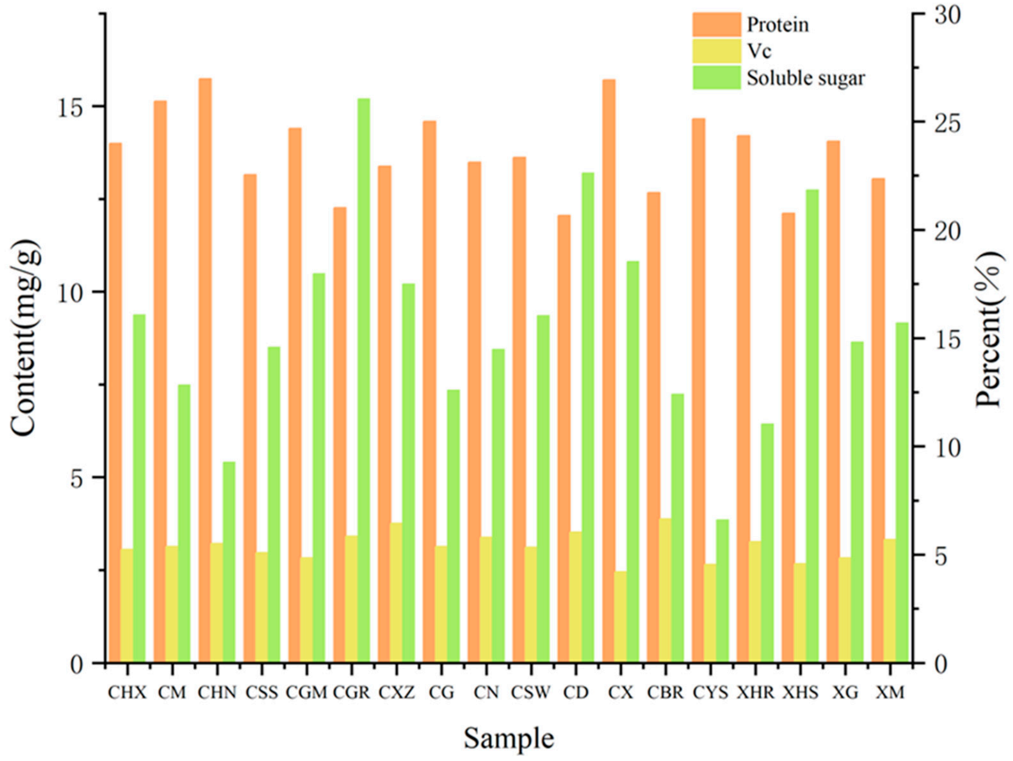

Figure S2. Changes of protein, vitamin C and soluble sugar in different dried capsicum. (Protein and Vc are represented by mg/g on the primary Y-axis and Soluble sugar by % on the secondary Y-axis.)
