# Supplementary material for: Analysis of Volatile and Non-Volatile Components of Dried Chili Pepper (Capsicum annuum L.)
Source: Foods. 2025 Feb 20;14(5):712. doi: 10.3390/foods14050712 (PMC11898792; doi:10.3390/foods14050712)
Supplement: Supplementary file 1 [file foods-14-00712-s001.zip › foods-3456206-Supplementary tables.pdf]

Table S1 Determination of volatile organic compounds peak area of different varieties of dried chili pepper by GC-TOF MS.

Table S1 Volatile organic compounds peak area measured by GC-TOF MS.

	CBR	CSW	XHR	XG	XHS	CM	CHN	CXZ	CGR	CYS	XM	CN	CGM	CSS	CD	CX	CHX	CG
Alcohols（62）																		
methyl mercaptan	58.17	131.21	68.43	139.51	59.73	132.11	288.58	51.15	126.72	303.76	199.01	203.28	151.28	208.59	116.67	161.13	82.14	73.31
methanol	79.86	99.01	204.04	178.59	72.42	109.53	282.35	97.05	52.72	163.36	104.88	166.19	54.46	60.63	47.56	95.44	112.42	115.77
ethanol	3649.11	4097.41	3326.65	3519.1	2708.94	3980.34	5240.12	2844.68	3065.37	4651.96	3545.88	3554.68	3812.81	3853.57	3646.72	3063.73	3178.38	2872.36
sec-butyl alcohol	-	-	4.27	-	-	0.53	-	0.43	-	0.46	-	-	-	-	-	-	-	0.17
isobutyl alcohol	10.49	12.92	8.45	15.29	20.42	-	14.01	3.73	9.6	54.33	-	23.69	15.83	12.49	13.18	13.14	11.97	2.45
3-methyl-2-butanol	-	-	5.18	-	-	-	-	-	1.17	35.02	1.36	2.3	-	2.08	-	-	-	24.17
3-pentanol	-	-	1.92	-	-	-	0.35	0.91	-	0.71	0.51	0.52	-	-	-	0.45	1.67	-
n-butyl alcohol	-	-	-	-	4.11	-	-	-	-	-	-	2.43	-	-	-	-	1.19	-
1-penten-3-ol	65.47	11.35	221.81	68.43	592.95	45.42	315.94	156.92	59.58	102.1	22.9	62.7	22.51	25.09	19.4	105.66	112.66	16.87
cineole	115.32	1091.76	10.83	-	2.24	858.13	7163.35	107.59	92.73	236.73	83.67	108.11	89.19	40.49	243.07	413.68	549.55	16.92
isoamyl alcohol	-	256.14	261.87	110.41	-	463.76	-	-	-	251.1	180.55	459	185.82	170.96	239.81	-	284.3	174.67
ethylidene diacetate	-	4.32	-	-	-	-	-	-	-	9.34	-	-	-	-	-	-	6.53	-
n-pentyl alcohol	-	416.47	312.03	287.17	107.69	465.55	441.91	-	478.81	342.06	400.79	385.33	342.47	377.52	296.95	251.17	330.42	241.85
4-methyl-1-pentanol	95.26	810.89	503.66	279.42	-	1414.34	363.31	44.89	68.94	710.07	669.89	1173.64	761.28	527.99	209.48	176.52	525.24	234.65
2-penten-1-ol, (2z)-	57.78	-	163.85	64.21	467.1	-	51.83	115.04	60.99	66.21	-	-	-	-	-	86.8	70.52	20.08
2-heptanol	15.36	-	-	17.19	-	-	-	13.97	1.21	54.79	1.45	2.36	1.39	-	-	-	-	-
1-hexanol	139.17	195.25	568.99	336.7	360.55	173.61	315.04	221.57	116.39	283.87	274.25	495.6	137.13	182.91	115.36	281.44	207.02	205.64
6-methylheptan-2-ol	-	-	-	-	-	-	-	-	0.79	-	-	-	-	-	1.42	-	-	0.32
3-hexenol	40.43	12.63	117.88	80.66	25.41	101.65	91.49	40.25	20.28	97.03	50	168.91	54.01	-	29.15	35.02	50.04	54.99
3-penten-1-ol, 4-methyl-	-	-	-	-	-	-	-	-	-	-	-	-	-	-	-	-	48.29	-
3-octanol	1.29	-	-	6.8	-	-	13.39	-	-	0.93	-	0.75	4.29	-	-	10.9	0.65	0.23
ethylene glycol mono-n-butyl ether	4.55	-	1.41	-	-	3.74	-	13.4	17.22	22.11	3.57	8.06	2.5	-	6.4	25.13	0.31	1.7
1-hexanol, 4-methyl-	-	-	53.87	-	-	-	-	-	-	-	-	51.81	-	16.6	3.41	4.68	20.8	-

	CBR	CSW	XHR	XG	XHS	CM	CHN	CXZ	CGR	CYS	XM	CN	CGM	CSS	CD	CX	CHX	CG
p-mentha-2,8-dien-1-ol, (z)-	-	-	-	-	-	-	564.3	-	-	-	-	-	-	-	-	-	-	-
linalool oxide, (z)-	78.95	-	45.85	-	48.42	110.05	195.37	51.24	9.43	117.59	-	80.07	-	-	-	66	-	-
linalool	1688.79	1462.6	30.87	13.96	20.66	2234.58	4005.35	629.42	2947.18	1842.31	43	1189.09	227.32	363.58	660.86	580.92	366.75	416.51
1-octen-3-ol	20.27	11.08	113.89	63.36	141.5	36.32	131.53	40.11	34.13	40.5	41.35	26.15	21.59	44.6	28.48	29.19	28.43	11.45
1-heptanol	19.98	7.73	48.98	3.09	148.49	15.38	18.28	51.1	68.56	12.76	12.04	-	14.48	21.36	11.85	27.71	27.93	-
sulcatol	-	-	-	-	16.47295	-	-	-	-	-	-	-	-	-	-	-	-	-
2-ethyl-1-hexanol	-	-	-	-	-	-	-	-	-	-	-	-	-	389.39	-	56.66	-	174.85
2-decanol	-	22.79	-	-	-	21.36	-	-	-	75.05	13.41	16.72	9.81615	12.6	-	3.74	-	3.65
propylene glycol, (s)-	-	58.48	-	-	70.99	-	10.83	10.25	-	71.27728	9.48	-	-	-	40.96	6.04	-	20.63
isopropanol	-	21.27	-	-	-	-	-	-	-	-	10.02	-	-	-	-	-	-	-
1-methylcycloheptan-1-ol	135.12	42.78	231.49	541.35	793.04	111.33	-	140.86	103.02	105.03	-	125.16	49.58	57.34	-	122.16	81.85	-
4-terpineol, (-)-	-	-	0.24	-	-	55.69	-	10.81	-	29.64	3.07	14.41	-	-	-	23.15	21.85	-
4-terpineol	26.39	46.46	-	-	-	51.15	1628.31	-	37.68	25.42	1.97	12.23	1.78	2.36	25.82	-	13.47	28.02
ethylene glycol	3.48	2.65	1.39	13.56	2.94	3.32	-	1.40	0.81	3.63	5.92	7.31	5.62	4.28	1.95	1.35	2.16	0.52
4-isopropylcyclohexanol	-	-	-	-	-	-	145.20	-	-	-	-	-	-	-	-	-	-	-
furfuryl alcohol	21.86	29.10	24.55	2.73	2.86	15.70	11.23	6.13	35.70	11.03	3.36	11.75	19.42	32.96	58.20	5.94	8.47	64.03
alpha-terpineol	11.21	32.21	-	-	-	39.15	285.56	5.94	26.90	-	-	-	12.16	12.28	14.68	-	-	-
5-methylfurfuryl alcohol	23.17	21.86	20.58	-	18.55	51.44	-	10.93	53.75	5.89	2.83	35.77	61.85	49.92	44.69	-	26.88	56.00
2-phenylisopropanol	1.09	0.12	-	5.25	7.76	5.48	1.09	3.47	0.87	3.36	0.14	0.70	35.06	41.26	7.85	6.19	1.15	2.60
myrtenol, (-)-	0.52	-	-	-	-	-	10.12	-	4.23	-	-	-	-	-	-	-	-	-
p,alpha-dimethylbenzyl alcohol	0.71	3.34	6.43	4.54	1.07	237.56	0.73	2.96	1.82	2.53	0.92	2.31	4.23	6.59	3.27	5.54	2.06	2.54
1-phenylethanol, (r)-	-	-	-	19.21	-	16.73	-	5.14	-	13.34	4.56	7.58	-	-	-	4.89	3.38	-
carveol, trans-(+/-)-	-	-	-	-	-	0.60	21.49	-	-	-	-	-	-	-	-	-	-	-
p-cymen-8-ol	22.14	11.04	-	-	-	19.43	62.45	24.72	20.44	11.19	-	11.92	24.47	43.83	24.78	27.41	-	7.79
2-methyl-4-penten-2-ol	-	-	-	-	-	66.36	-	-	-	48.84	5.18	-	50.18	-	23.97	-	-	-

	CBR	CSW	XHR	XG	XHS	CM	CHN	CXZ	CGR	CYS	XM	CN	CGM	CSS	CD	CX	CHX	CG
pinacol	6.19	22.11	11.75	42.94	-	19.67	37.66	-	-	52.19	15.91	26.37	0.00	10.47	19.73	-	4.39	8.40
benzyl alcohol	28.6	38.68	66.53	55.64	21.48	98.62	82.12	22.63	23.69	113.38	36.03	87.07	95.30	66.23	26.92	33.23	38.78	72.08
2-(2-hydroxypropoxy)propan-1-ol	11.35	5.04	-	9.62	21.41	6.41	45.20	1.32	9.11	1.84	-	9.37	6.27	6.33	1.26	1.46	1.45	1.57
2-phenylethanol	24.96	24.30	20.13	32.54	14.04	47.68	47.82	15.41	34.18	25.27	16.56	33.07	32.59	28.74	34.49	17.75	15.83	38.46
(E)-2,6-Dimethylocta-3,7-diene-2,6-diol	3.05	-	-	-	-	-	-	-	-	-	-	-	-	-	0.25	-	15.39	0.31
dihydro-beta-ionol	1.5	-	2.22	2.44	-	1.19	6.98	-	-	2.17	0.24	3.55	0.15	0.95	1.07	1.01	1.12	5.43
1-undecanol	-	-	-	-	-	-	-	-	-	-	-	-	-	-	-	-	-	0.43
perilla alcohol	-	-	-	-	-	-	13.04	-	-	-	-	-	-	-	-	-	-	-
3-phenylpropanol	0.28	0.28	-	0.37	0.05	0.24	-	-	0.06	-	0.06	-	0.08	-	-	-	-	-
nerolidol	61.45	52.53	48.53	46.15	5.21	12.29	29.90	5.56	9.45	53.43	62.14	35.47	11.44	9.92	15.86	7.09	5.01	8.42
cumic alcohol	-	0.06	0.06	0.50	-	9.93	24.96	0.23	0.10	0.07	-	0.05	0.03	-	0.44	0.04	0.43	0.03
cedrol	-	-	-	-	-	-	-	-	-	-	-	-	0.55	-	2.91	-	-	1.58
bisabolol	0.3	-	-	-	-	-	-	-	-	-	-	-	-	-	0.15	-	-	-
(-)-drimenol	-	-	-	-	-	0.79	-	-	-	-	-	-	1.62	2.22	-	0.46	0.97	-
Aldehydes（50）																		
propionaldehyde	387.70	-	442.53	310.25	195.82	2.88	642.29	-	384.56	740.67	-	-	1.47	-	-	240.86	417.78	3.67
isobutyraldehyde	544.85	602.42	781.70	89.37	698.92	661.77	203.51	454.10	438.92	795.77	713.02	194.00	518.91	545.74	507.36	158.41	153.02	448.44
methacrolein	5.62	1.31	-	0.44	22.31	-	0.53	1.55	1.01	10.23	-	154.00	0.89	1.05	4.97	1.45	-	0.97
2-methylbutyraldehyde	50.12	63.51	148.90	72.96	149.31	42.30	34.95	72.16	75.56	61.18	101.08	68.48	41.28	60.00	90.00	36.51	43.85	171.87
3-methylbutanal	199.38	249.21	365.26	238.63	553.21	186.01	68.12	261.92	347.11	171.04	295.72	239.06	169.96	227.97	351.63	101.93	126.06	388.37
pentanal	77.52	109.33	125.02	117.56	249.20	121.03	100.26	90.52	114.32	143.87	69.13	90.12	89.66	123.07	62.76	89.58	93.83	66.42
hexaldehyde	529.43	258.02	1707.54	595.82	2925.21	648.31	649.10	870.61	1035.05	699.34	621.76	656.72	522.62	1077.94	414.84	846.51	918.03	368.13
trans-2-pentenal	-	-	-	-	118.58	-	-	-	-	-	-	-	-	-	-	-	-	-
2-butenal, 2-ethyl-	-	-	-	1.42	-	-	-	-	-	-	-	-	-	-	-	-	2.39	-

	CBR	CSW	XHR	XG	XHS	CM	CHN	CXZ	CGR	CYS	XM	CN	CGM	CSS	CD	CX	CHX	CG
2-methyl-2-pentenal	3.06	4.43	6.60	1.47	4.86	-	-	3.86	4.21	-	5.94	-	-	2.75	6.08	3.94	2.41	2.11
n-heptanal	68.42	42.79	147.33	83.01	257.42	28.94	20.51	93.64	103.14	33.52	51.07	63.95	36.05	80.77	39.30	69.14	51.51	42.56
senecialdehyde	1.37	0.49	4.29	0.87	19.83	-	-	1.46	1.20	0.71	0.27	0.78	0.43	0.65	1.50	1.11	1.40	1.37
trans-2-hexenal	124.89	-	266.08	166.07	379.41	68.93	15.80	175.51	174.59	-	175.13	450.05	276.07	176.63	291.57	249.41	61.69	150.46
4-heptenal, (4z)-	-	-	17.91	-	125.72	-	-	-	-	-	-	-	-	-	-	-	-	-
octylaldehyde	47.89	75.76	162.94	57.32	188.31	68.87	23.02	25.05	61.93	32.16	27.10	57.01	32.56	87.53	39.35	46.27	54.08	27.86
2-ethyl-3-propylacrolein	-	15.09	-	-	-	-	-	-	-	-	-	-	0.72	6.50	2.32	-	-	-
nonanal	78.55	43.28	75.84	36.58	152.10	82.04	98.39	55.83	108.48	51.82	82.00	54.79	54.11	60.82	48.21	51.57	43.39	37.43
4-thiapentanal	-	128.43	113.67	170.37	133.53	232.84	369.03	117.68	-	207.13	138.77	-	-	-	138.63	-	48.33	87.85
furfural	98.34	117.68	32.90	9.70	14.52	47.44	22.76	16.98	217.03	22.44	59.96	31.71	64.81	84.01	131.64	10.84	29.17	215.60
2,4-heptadienal	19.48	-	46.72	6.41	147.02	15.71	18.23	50.18	67.62	10.89	14.58	12.62	14.58	20.22	11.85	31.09	27.78	12.16
decaldehyde	6.22	-	-	-	3.36	-	-	4.89	20.17	-	-	-	-	-	-	3.19	1.02	-
benzaldehyde	96.64	40.53	199.51	208.77	336.99	294.65	115.17	155.18	146.17	76.18	27.61	46.55	42.68	127.51	92.57	101.56	84.52	107.67
2-nonenal	-	11.66	-	-	-	16.52	-	-	12.02	-	15.48	12.41	11.48	19.30	7.56	-	-	14.17
5-methyl-2-furfural	30.63	107.25	48.11	-	-	182.50	-	5.23	110.75	1.70	-	18.76	121.87	137.03	106.11	110.09	12.15	16.33
trans-2,cis-6-nonadienal	129.11	-	-	-	62.91	-	-	33.55	50.98	-	-	-	-	-	16.50	-	-	5.99
2,6-nonadienal, (2e,6e)-	-	-	-	-	-	-	-	27.57	43.77	-	-	-	-	-	10.81	-	-	-
beta-cyclocitral	79.54	49.99	109.10	241.62	362.31	58.73	44.60	85.16	67.03	50.03	-	48.45	31.75	36.44	22.51	67.59	35.24	23.00
myrtenal	0.64	-	-	-	-	-	29.95	-	1.36	0.26	-	-	-	-	-	-	-	-
phenylacetaldehyde	55.69	71.30	91.48	33.32	132.49	74.31	82.61	61.95	82.74	52.46	127.85	58.61	74.06	102.73	99.17	35.16	27.47	117.76
safranal	169.94	-	237.36	743.04	36.29	-	-	91.00	86.64	197.06	-	-	-	-	42.24	118.62	101.84	125.47
benzaldehyde, 2-ethyl-	-	1.66	-	-	-	-	-	3.50	-	-	-	-	-	-	-	-	-	-
2,5-dimethylbenzaldehyde	3.10	-	1.13	4.62	10.70	-	-	-	4.74	2.26	-	-	-	-	-	-	3.82	-
2,6-dimethylbenzaldehyde	-	-	-	4.71	4.26	-	-	3.90	-	-	-	-	-	-	-	-	-	-
3,3-diethoxypropyne	-	2.38	-	-	-	-	-	-	-	-	-	-	1.13	-	-	-	0.83	-

	CBR	CSW	XHR	XG	XHS	CM	CHN	CXZ	CGR	CYS	XM	CN	CGM	CSS	CD	CX	CHX	CG
cuminaldehyde	5.99	23.38	4.48	39.36	8.88	93.70	90.59	15.50	21.91	10.80	10.23	18.64	12.04	11.62	15.48	3.68	2.71	16.21
5-methyl-2-thiophenecarboxaldehyde	-	2.51	20.59	1.34	-	7.57	-	-	-	-	0.51	-	0.39	1.47	1.08	0.91	-	0.75
2-trans-4-trans-decadienal	-	-	-	-	7.67	-	-	-	-	-	-	-	-	-	-	-	-	15.12
3,4-difluorobenzaldehyde	-	-	-	-	-	-	-	-	-	-	-	2.14	1.60	1.60	-	-	-	-
2-phenyl-2-butenal	0.53	1.44	1.36	0.85	-	1.31	0.21	-	1.35	0.45	1.07	0.68	1.45	1.94	1.42	0.22	0.41	1.64
pentadecanal	-	-	-	-	-	-	-	-	-	-	7.36	-	3.30	1.09	-	-	-	0.75
pyrrole-2-carboxaldehyde	4.78	5.42	6.82	4.46	3.73	-	1.19	0.60	6.72	1.36	-	2.34	1.50	1.62	4.28	0.43	2.28	8.64
p-anisaldehyde	1.37	3.49	1.10	-	-	-	-	1.37	2.74	-	-	2.02	-	2.82	3.37	1.38	0.56	2.41
trans-cinnamaldehyde	2.53	1.03	1.07	-	2.53	-	-	2.87	2.96	2.15	0.35	0.53	-	-	2.14	3.34	2.75	-
undecanal	-	-	-	-	-	-	5.13	-	-	-	-	-	-	-	-	-	-	-
1-methyl-1h-pyrrole-2-carboxaldehyde	1.45	2.56	0.36	-	-	-	-	-	2.66	-	-	0.28	0.63	0.86	0.49	-	-	1.89
4-Hydroxy-2,6,6-trimethylcyclohex-1-enecarbaldehyde	2.65	1.09	1.74	3.13	3.46	1.99	6.59	2.63	3.54	3.16	-	1.47	1.08	1.20	1.15	1.76	1.26	0.68
5-hydroxymethyl-2-furfuraldehyde	3.14	1.19	0.78	0.51	0.41	1.24	0.89	0.44	6.17	1.46	0.39	0.61	1.66	2.05	3.99	0.99	0.97	4.63
vanillin	0.47	1.29	-	-	-	0.49	-	-	-	-	0.74	0.51	0.31	-	-	-	-	0.37
isovanillin	-	0.58	0.68	-	-	-	-	-	-	1.09	-	0.17	-	-	-	-	-	0.35
metaldehyde	-	-	6.33	-	-	523.73	-	15.83	-	8.88	378.31	-	-	-	-	18.23	-	407.05
Ketones（68）																		
acetone	111.87	82.85	297.59	-	324.35	2.99	-	-	-	-	80.82	-	-	-	-	-	-	1.97
methyl ethyl ketone	20	23.77	61.79	52.74	18.34	25.41	137.26	22.74	15.57	66.6	28.05	-	14.29	16.09	13.57	26.71	46.67	37.35
diacetyl	19.28	21.65	22.71	222.88	416.08	15.62	13.54	12.46	11.62	16.06	24.01	19.47	9.89	8.97	19.9	15.16	19.41	10.73
deiodoamiodarone	-	-	148.94	-	27.9	-	-	-	12.49	128.62	-	-	-	-	-	-	-	-
2-methylpentan-3-one	4.44	5.58	13.08	6.48	-	14.04	24.98	6.46	4.02	22.3	11.87	9.6	9.36	9.21	5.4	-	4.48	4.12

	CBR	CSW	XHR	XG	XHS	CM	CHN	CXZ	CGR	CYS	XM	CN	CGM	CSS	CD	CX	CHX	CG
1-pentene-3-one	36.23	9.88	22.5	13.34	75.63	-	22.13	36.66	18.38	44.68	11.04	23.68	12.84	7.78	15.55	58.12	40.37	12.62
3-penten-2-one (trans)	-	-	2.09	-	-	-	-	-	-	-	-	-	-	-	-	-	-	-
5-methylhexan-3-one	0.33	1.05	2.08	-	-	-	-	-	-	2.01	-	0.69	1.77	1.03	-	-	0.71	0.77
3-penten-2-one	-	-	108.07	-	-	-	-	177.05	-	-	-	-	-	-	-	-	-	-
2-methyl-4-heptanone	-	-	-	-	-	-	-	-	-	10.13	-	-	-	-	-	-	-	0.17
3-heptanone	-	4.59	6.55	5.28	5	2.86	8.38	3.86	4.21	6.77	5.94	2.65	3.02	-	6.08	3.09	2.41	2.11
2-heptanone	7.48	20.39	39.84	52.49	25.37	13.95	0.21	10.46	6.45	14.53	19.08	12.97	9.63	33.25	8.95	9.6	12.82	20.57
5-methyl-2-hexanone	7.11	-	-	-	-	-	-	-	-	-	-	-	-	-	-	-	-	-
2-isooctanone	-	-	35.05	27.69	124.02	-	-	38.24	20.88	24.37	-	-	-	-	-	-	-	-
3-hexen-2-one, 5-methyl-	-	-	-	-	-	-	-	-	-	-	3.94	8.16E-06	-	-	-	-	-	-
3-octanone	204.32	-	136.05	178.64	-	193.02	265.09	169.13	-	205.16	163	148.05	177.01	-	-	200.72	127.01	124.28
acetoin	97.3	216.15	219.57	542.25	288.08	-	72.99	75.59	113.18	70.09	100.93	103.19	62.51	67.96	-	65.45	71.75	50.83
cyclohexanone	97.59	-	-	-	5.32	-	-	129.64	125.06	220.34	-	77.46	145.06	100.17	116	202	-	48.96
2-octanone	22.9	10.46	53.35	78.63	74.06	24.87	71.39	105.61	18.04	77.85	159.25	72.73	17.41	17.86	23.09	55.06	20.87	17.19
acetol	59.74	106.41	169.09	69.64	187.09	75.61	25.79	57.15	59.66	53.35	66.26	61.21	61.92	89.25	55.88	65.94	57.14	97.61
3-hepten-2-one	5.29	32.92	61.81	36.82	22.84	14.73	19.15	21.83	3.28	33.87	9.72	13.56	7.74	13.58	7.35	12.78	34.76	16.93
2-octen-4-one	-	37.97	61.28	-	-	-	-	-	-	30.54	-	12.72	-	-	-	-	-	-
methylheptenone	97.02	51.08	360.07	220.4	1000.28	107.49	155.19	150.82	107.59	120.82	71.47	90.27	64.44	54.12	45.84	115.81	94.59	50.34
cyclopentenone	-	-	-	-	-	-	-	-	1.38	-	-	-	-	4.51	1.35	-	2.21	4.86
cyclohexanone, 3,3-dimethyl-	3.83	-	38	24.1	78.51	-	4.23	10.45	1.13	11.7	-	2.75	-	-	-	3.03	6.84	-
4-hydroxyacetophenone	-	-	-	-	-	-	-	-	-	80.22	-	-	-	-	-	-	-	-
isophorone	16.15	-	27.48	27.42	98.1	-	-	24.6	22.01	7.9	-	4.08	-	-	-	12	-	-
3-octen-2-one	-	-	-	527.91	37.82	1153.08	141.57	46.34	29.14	-	76.79	389.27	-	-	149.88	6	28.88	148.91
beta-thujone	-	48.21	-	-	-	-	762.28	-	-	-	-	-	-	-	-	-	-	-

	CBR	CSW	XHR	XG	XHS	CM	CHN	CXZ	CGR	CYS	XM	CN	CGM	CSS	CD	CX	CHX	CG
d-isothujone	5.49	-	-	-	-	14.38	322.1	-	-	15.58	-	-	-	-	20.03	-	-	182.01
2,3-dimethylacetophenone	4.73	-	126.99	64.05	143.21	-	-	7.99	3.92	-	-	6.24	5.75	12.34	7.49	4.02	6.08	5.15
acetoxyacetone	7.43	3.18	11.67	8.1	-	-	-	1.49	7.42	-	0.11	0.72	1.86	2.22	1.72	0.68	5.62	9.83
menthone	-	3.46	-	-	-	-	-	-	-	-	-	-	-	-	-	-	-	-
2,3-dimethyl-2-cyclopentenone	25.71	29.66	163.02	236.27	-	52.09	33.43	101.9	31.93	37.21	33.9	28.74	36.17	40.86	21.52	56.5	28.62	28.82
4-cyclopentene-1,3-dione	6.12	3.05	15.97	-	-	1.21	8.84	1.72	5.18	128.01	2.83	59.97	140.32	45.43	18.05	2.07	28.04	49.39
ethanone, 1-(2-methyl-1-cyclopenten-1-yl)-	-	12.01	-	-	-	-	-	-	-	50.02	-	-	-	16.53	-	-	-	20.63
3,5-heptadien-2-one, 6-methyl-	101.25	44.47	173.04	-	68.9	18.32	-	48.74	45.26	46.65	-	34.21	22.59	18.73	24.35	47.02	30.81	17.66
d-dihydrocarvone	-	-	-	-	-	-	-	-	-	-	-	-	-	-	7.26	-	-	-
acetophenone	174.56	151.38	-	-	35.67	-	311.21	93.66	85.74	-	-	22.72	-	28.78	41.02	19.14	12.61	107.68
4-hydroxy-4-methyl-5-hexenoic acid gamma lactone	14	14.36	12.21	26.46	6.25	10.56	15.12	7.81	12.37	18.05	4.69	11.52	9.66	11.62	25.72	7.96	5.32	18.95
1-methyl-2-pyrrolidinone	55.6	98.99	122.32	51.25	47.57	83.29	88.9	44.18	53.42	119.27	58.23	57.02	58.73	60.17	52.89	41.93	55.33	85.51
ketoisophorone	24.51	27.78	59.41	44.01	138.24	27.21	40.69	35.44	47.02	34.52	20.52	16.25	22.05	25.05	11.74	34.18	30.58	6.79
2,5-hexanedione	10.14	-	149.11	7.65	-	-	-	40.43	92.68	-	-	-	-	-	11.58	7.56	65.68	-
2(5h)-furanone, 3-methyl-	0.88	4.16	0.91	3.71	1.48	1.69	16.43	-	2.28	2.88	-	1.26	-	2.04	2.95	-	-	2.08
2(5h)-furanone, 4-methyl-	0.77	3.78	-	3.94	1.29	-	-	-	1.47	2.43	-	1.3	-	2.4	-	1.06	0.62	-
carvone	1.05	8.79	-	-	-	-	6.05	-	3.87	2.11	-	-	-	-	10.01	-	-	-
2(5h)-furanone	23.69	36.55	30.52	-	2.61	79.19	-	15.44	38.64	-	-	24.82	36.01	62.6	19.26	-	-	33.57
p-methyl acetophenone	6.99	22.9	13.89	9.62	4.28	78.31	42.87	12.55	12.58	58.19	30.28	28.21	25.19	27.33	22.72	7.63	5.77	34.57
3-methylacetophenone	7.26	-	11.83	7.29	4.25	-	45.49	15.05	2.88	51.37	-	27.82	-	24.51	-	7.16	-	10.44
1,4-cyclohexanedione, 2,2,6-trimethyl-	21.87	6.69	20.21	9.83	7.49	4.08	8.96	3.72	5.88	28.33	2.47	18.62	15.93	-	3.69	17.97	11.75	-
cyclobutanone	-	-	1.85	-	11.84	1.25	-	-	-	-	-	-	-	-	-	-	-	-

	CBR	CSW	XHR	XG	XHS	CM	CHN	CXZ	CGR	CYS	XM	CN	CGM	CSS	CD	CX	CHX	CG
2-tridecanone	-	0.47	-	-	0.42	1.31	-	-	1.24	-	-	1	1.61	2.49	1.28	1.41	1.43	0.14
dihydro-alpha-ionone	2.34	-	1.47	3.46	-	6.38	-	-	-	5.9	1.03	-	1.54	2.49	-	-	0.23	0.83
dihydro-beta-ionone	14.57	10.85	11.93	19.01	9.65	13.66	37.87	8.49	8.86	11.11	4	22.03	6.57	13.7	9.7	8.59	5.62	15.99
alpha-ionone	30.11	12.92	18.34	51.36	21.3	45.23	11.15	11.25	13.53	21.84	15.08	11.63	17.31	22.84	11.38	11.29	6.6	7.77
gamma-lonone	-	-	-	-	-	-	14.42	-	-	-	-	-	-	-	-	-	-	-
geranylacetone	36.6	3.75	33.64	51.56	208.04	22.21	42.83	22.82	30.69	5.74	-	10.57	-	5.27	2.89	27.3	19.05	14.32
beta-ionone	34.93	-	34.91	100.04	103.49	104.56	104.31	22.26	24.39	50.72	26.36	59.94	43.77	39.11	14.2	26.23	14.68	18.44
trans-beta-ionone	60.33	25.04	-	187.19	187.19	-	-	-	-	-	14.04	-	43.65	51.54	-	-	-	-
2-furyl hydroxymethyl ketone	19.23	-	17.61	-	-	-	-	14.55	-	-	-	-	-	-	-	-	-	-
3-buten-2-one, 4-(2,2,6-trimethyl-7-oxabicyclo(4.1.0)hept-1-yl)-	18.19	-	16.92	44.02	48.26	51.06	25.72	13.71	17.71	14.16	-	14.16	12.48	44.29	32.73	17.16	10.22	30.63
2-pyrrolidone	30.93	13.59	13.51	37.47	9.69	9.97	18.1	11.69	24.14	20.48	5.34	6.55	2.44	7.5	9.2	10.14	10.61	11.34
benzoyl isopropanol	-	-	-	-	-	1.68	-	-	-	-	-	-	-	-	-	-	3.67	-
4-hydroxy-5-methyl-3(2h)-furanone	1.23	5.85	4.27	4.6	1.55	-	2.38	1.85	1.3	1.62	3.79	3.79	1.12	1.44	1.84	-	2.22	3.86
2,3-dihydro-3,5-dihydroxy-6-methyl-4h-pyran-4-one	77.52	75.61	63.5	21.83	5.7	23.15	15.57	21.95	76.28	16.94	5.5	50.11	42.96	39.95	66.5	15.52	18.98	196.11
3-ethyl-4-methyl-1h-pyrrole-2,5-dione	6.39	6.06	5.01	7.48	4.48	5.72	2.65	6.17	6.49	3.18	1.31	4.23	4.35	4.4	4.74	3.24	3.55	10.68
oxacyclotetradecane-2,11-dione, 13-methyl-	-	-	1.27	2.2	-	1.09	-	-	-	-	-	2.92	-	-	-	-	-	-
2(3h)-furanone, dihydro-5-(hydroxymethyl)-	1.23	-	-	-	-	-	-	-	1.2	-	-	-	-	-	1.64	-	-	0.73
Alkenes（60）																		
1-methylcyclopentadiene	-	-	-	-	224.31	-	-	-	-	-	-	-	-	-	-	-	-	-
propylene	-	-	12.89	-	21.25	-	-	-	-	-	-	-	-	-	-	-	-	-

	CBR	CSW	XHR	XG	XHS	CM	CHN	CXZ	CGR	CYS	XM	CN	CGM	CSS	CD	CX	CHX	CG
1,2,4,4-tetramethylcyclopentene	2018.44	-	1746.15	1915.79	1563.23	-	2565.14	1504.2	-	2354.39	-	1837.83	2028.33	-	1590.71	3747.53	1551.7	-
alpha-pinene	77.79	68.17	-	-	-	106.58	1782.09	-	33.41	123.39	42.86	78.03	46.21	28.93	51.53	124.88	132.97	25.42
(-)-alpha-pinene	-	-	-	-	-	-	-	-	-	-	41.97	-	-	-	-	-	-	-
bicyclo(3.1.1)hept-2-ene, 3,6,6-trimethyl-	-	-	-	-	17.93	-	-	48.36	36.87	-	-	-	-	-	-	-	-	-
alpha-pinene, (+)-	-	-	104.25	101.66	-	-	-	-	-	-	-	-	-	-	-	-	-	-
camphene	-	0.66	1.86	-	0.32	-	20.85	-	0.17	-	-	0.19	-	-	-	0.23	0.73	0.6
beta-pinene	11.42	10.21	-	-	-	34.75	-	-	-	18.1	-	-	-	1.06	-	-	-	6.91
(-)-beta-pinene	11.9	19.27	-	7.72	-	43.16	334.01	6	5.68	37.15	2.14	16.7	9.06	1.73	13.05	11.31	36.17	0.45
carene	9.81	-	-	-	-	4.82	10.32	-	5.45	20.17	-	110.69	-	6.47	-	15.02	0.09	-
3-carene, (+)-	-	27.95	-	-	-	-	-	7.71	-	-	6.96	-	-	-	14.43	-	-	5.36
alpha-phellandrene	66.97	29.12	221.81	67.02	145.65	53.07	341.28	160.47	61.62	107.96	6.69	62.7	22.94	25.09	30.19	107.8	117.28	18.22
myrcene	449.79	1339.25	247.33	136.98	288.59	1696.75	12327.61	898.49	497.63	1379.6	562.34	491.08	373.35	255.44	403.5	588.89	664.54	176.02
1,4-diacetoxy-2-butene	12.11	-	-	-	-	-	-	-	-	10.35	-	-	-	14.25	13.39	21.06	8.03	-
alpha-terpinene	177.62	504.19	-	-	-	-	1608.81	-	-	130.96	-	-	-	-	-	-	-	-
(d)-limonene	-	-	-	-	330.68	4798.17	15201.43	1925.53	-	-	1338.31	1627.75	980.17	893.38	1112.19	-	1781.02	478.77
limonene	1465.62	4544.74	402.09	335.57	329.99	4774.80	10456.01	2001.94	1596.19	4902.5	1316.11	1633.46	-	-	-	1942.37	1845	-
sabinene	187.87	518.58	32	54.83	37.8	834.56	-	296.37	219.48	496.81	208.11	193.34	140.22	114.05	119.15	207.53	298.63	69.24
beta-phellandrene	21.87	523.46	-	53.19	-	792.37	5660.3	-	224.51	249.12	206.31	53.03	-	108.53	-	-	311.02	-
gamma-terpinene	55.99	145.38	42.91	27.18	-	178.31	1434.76	38.64	47.88	124.78	48.33	42.62	33.09	23.1	52.06	50.79	58.99	22.74
cyclooctatetraene	2641.09	-	-	-	-	-	-	-	4486.32	-	5594.28	-	-	-	-	-	1111.46	-
alpha-ocimene	-	-	-	-	-	1189.73	-	1981.65	776.25	1112.76	-	951.49	-	744.17	1032.08	954.61	-	774.05
beta-ocimene, (3z)-	-	-	-	-	605.28	-	-	-	-	-	1046.14	939.48	-	727.56	-	-	-	-
beta-ocimene	1942.53	816.01	702.3	1455.01	626.87	1196.67	3061.43	1977.66	-	-	1041.84	-	1605.36	-	648.28	-	1110.61	960.72
styrene	1.86	-	-	-	-	-	-	1.17	-	-	1.85	1.03	0.39	0.81	0.94	1.23	1.21	-

	CBR	CSW	XHR	XG	XHS	CM	CHN	CXZ	CGR	CYS	XM	CN	CGM	CSS	CD	CX	CHX	CG
terpinolene	-	159.31	-	8.51	-	61.28	685.59	-	-	-	9.5	-	-	-	-	-	21.75	-
alloocimene, (4e,6e)-	-	-	-	-	-	-	-	-	-	-	-	-	-	-	21.9	-	-	-
1,3,5,5-tetramethyl-1,3-cyclohexadiene	38.81	-	-	-	-	-	-	-	62.67	-	-	-	-	-	-	24.41	-	-
alloocimene, (4e,6z)-	36.56	71.71	108.51	17.66	-	109.60	432.62	38.37	64.91	-	-	-	-	-	37.48	36.41	45.58	73.82
alloocimene	80.41	-	-	-	-	-	152.09	66.98	-	-	105.12	-	-	-	-	50.99	52.35	57.76
cosmene	238.69	-	-	335.47	-	-	566.45	170.62	-	-	-	-	221.03	227.04	-	-	142.71	-
sabinene hydrate	-	-	-	-	-	-	3311.07	61.68	138.64	-	-	-	-	-	70.03	-	-	-
copaene	-	-	1.31	-	-	-	-	-	-	5.85	22.23	-	-	-	-	-	-	-
2,4-octadiene	-	-	-	-	-	-	-	-	-	-	-	-	-	-	-	47.52	-	-
longicyclene	20.66	13.35	62.73	183.37	-	-	-	9.59	19.7	52.09	26.27	10.96	0.63	3.31	-	0.79	-	1.00
vinyl fluoride	6.93	-	7.06	-	-	-	-	-	64.99	-	2.54	34.38	-	-	-	-	-	-
1-pentadecene	106.01	167.15	-	-	-	-	31.8	-	-	16.63	57.88	145.91	167.79	139.16	-	-	-	113.23
valencene	-	-	-	-	-	-	-	-	-	52.72	43.14	-	-	-	-	-	-	-
(+)-cyclosativene	49.42	75.26	91.38	60.88	-	13.48	117.91	0.09	0.17	270.96	-	-	11.32	9.6	2.24	4.55	4.51	-
3,7-dimethyloct-1-ene	-	-	-	-	-	-	-	-	-	-	4.09	-	-	-	-	-	-	-
1-tetradecene	-	-	-	-	-	-	-	-	-	6.38	-	-	-	-	-	-	-	1.66
n-hexadec-1-ene	-	-	-	-	-	10.26	-	-	-	6.09	-	-	14.86	-	-	-	-	-
(-)-beta-elemene	3.78	3.75	12.2	-	70.47	23.28	-	22.7	17.81	85.63	15.05	62.97	21.83	-	23.13	28.51	23.03	35.06
thujopsene	-	-	4.56	5.94	-	-	3.02	0.99	-	-	7.02	3.44	6.48	5.47	0.99	3.38	3.03	0.63
beta-farnesene	-	-	13.4	45.19	-	78.19	53.57	13.42	-	-	34.19	34.21	35.74	64.86	-	23.78	20.77	-
longipinene	-	-	-	-	-	3.71	1.67	-	-	-	-	1.41	4.13	12.24	-	0.2	1.13	0.87
eremophilene	3.53	22.07	-	19.81	17.27	-	79.47	-	56.69	14.32	5.22	-	-	-	-	23.78	25.81	-
Valerena-4,7(11)-diene	-	-	-	-	-	-	-	-	-	-	-	35.77	-	-	-	-	-	-
(+)-aromadendrene	65.61	249.88	133.46	371.11	-	7134.64	6859.59	39.98	71.12	134.37	83.72	4057.73	61.29	5215.64	44.17	2409.46	2479.02	-

	CBR	CSW	XHR	XG	XHS	CM	CHN	CXZ	CGR	CYS	XM	CN	CGM	CSS	CD	CX	CHX	CG
alloaromadendrene	-	-	-	-	-	50.88	-	42.53	-	-	-	-	5559.49	2810.62	-	9.27	0.78	2273.78
beta-caryophyllene	-	-	-	-	-	-	-	-	48.2	-	-	-	5372.27	-	47.19	-	-	-
1-heptadecene	-	-	-	-	-	-	-	-	-	-	1.62	-	-	-	-	-	-	-
beta-cedrene	-	-	-	-	-	11.23	17.33	-	-	-	-	8	8.94	7.49	-	0.84	3.1	2.1
sesquiphellandrene, (-)-beta-	-	1.01	-	-	-	0.95	15.82	-	-	-	-	7.05	8.52	-	-	-	0.23	2.53
(1beta,7beta)-cedr-8(15)-ene	-	-	-	-	-	12.19	-	-	-	-	-	6.71	8.7	7.12	-	3.69	2.85	2.63
acenaphthylene	-	-	-	-	-	-	-	-	-	-	-	0.7	-	-	1.93	0.26	0.14	1.26
o-cymene	-	102.58	34.52	67.11	49.87	308.58	2417.14	130.67	94.93	210.47	88.25	87.07	62.77	41.36	57.27	122.04	131.06	30.32
m-cymene	6.15	8.27	-	-	6.09	-	11.51	-	-	-	-	8.27	-	-	-	9.24	6.68	6.04
naphthalene	40.73	34.08	8.89	1065	15.89	-	103.74	8.93	7.74	16.53	6.1	-	5372.27	-	16.79	40.04	30.57	47.22
Esters（99）																		
methyl isocyanate	-	-	-	-	463.2	147.37	1414.36	-	-	-	-	-	-	524.09	-	-	-	-
benzoic acid, 4-propyl-, 4-pentylphenyl ester	278.19	-	-	-	-	-	-	-	-	-	-	-	-	-	-	-	-	-
vinyl formate	-	-	487.83	-	623.91	3780.96	-	-	-	-	-	-	-	-	-	-	-	857.83
methyl formate	-	-	-	1877.48	24.71	-	182.35	1082.97	-	-	1514.98	-	-	-	-	-	-	5.37
1,3-epoxypropane	-	-	-	469.97	-	2.03	377.11	584.98	150.2	8.4	-	547.07	1.6	-	2.32	-	-	2.99
1-propen-2-ol, acetate	-	-	-	-	-	543.69	-	-	-	-	-	-	500.39	-	-	-	-	-
methyl acetate	273.12	527.48	-	393.34	-	373.74	544.62	254.86	267.94	458.53	-	386.54	304.08	310.09	259.67	336.62	323.01	-
ethyl acetate	38	19.46	35.22	129.09	37.11	21.55	173.42	45.09	27.44	120.31	33.05	160.71	19.7	18.01	17.96	76.64	63.59	8.28
diazocarboxylic acid ethyl ester	-	-	-	-	-	-	-	-	57.32	-	-	-	-	-	-	-	-	-
butyl pyruvate	-	-	-	-	-	-	-	-	-	-	7.53	-	-	-	-	-	-	-
2-furanmethanol, tetrahydro-, acetate	-	-	-	-	-	296.79	13.62	-	13.6	-	-	-	-	-	-	-	-	-
vinyl propanoate	-	-	-	-	-	-	-	-	-	-	-	-	-	-	-	10.82	-	-

	CBR	CSW	XHR	XG	XHS	CM	CHN	CXZ	CGR	CYS	XM	CN	CGM	CSS	CD	CX	CHX	CG
methyl methacrylate	-	0.55	-	-	-	0.32	-	-	0.23	2.51	-	0.39	0.6	1.04	0.23	0.57	-	0.25
n-succinimidyl acrylate	-	-	-	-	-	-	-	413.65	-	-	-	-	-	-	-	-	-	-
vinyl acrylate	442.52	489.25	426.30	-	-	-	-	-	-	-	-	-	-	-	-	-	559.61	341.06
ethyl-2-methyl butyrate	-	-	-	1.56	-	-	-	1.01	2.27	7.91	2.36	2.36	1.91	0.64	1.91	0.11	0.56	0.69
isoamyl formate	-	-	2.16	-	0.42	-	-	-	-	-	-	1.29	-	-	-	-	-	-
n-butyl acetate	4.08	4.58	0.82	0.97	0.93	8.61	1.92	5.58	5.16	10.07	8.16	5.53	6.55	5.75	9.62	9.01	3.49	5.73
isoamyl acetate	-	15.1	-	-	-	-	-	-	66.09	87.41	30.39	18.85	8.74	44.79	15.55	-	56.32	28.52
ethyl valerate	-	202.15	135.23	266.25	64.97	235.71	-	364.93	-	247.84	337.36	150.3	187.23	-	-	-	-	166.09
n-butyl proprionate	0.18	-	-	-	-	-	-	0.34	0.56	2.85	4.29	-	-	-	0.23	0.62	0.83	-
isobutyl 2-methylbutyrate	-	-	-	54.4	24.96	-	-	-	-	-	-	-	-	-	-	-	-	-
methyl caproate	5.67	-	10.45	6.82	9.15	1.96	0.68	3.64	4.85	2.24	0.69	2	1.16	7.24	1.48	5.05	-	0.71
butyl butyrate	4.36	2.23	1.88	-	-	12.16	0.99	2.93	9.14	0.92	14.25	3.57	6.36	4.17	7.57	10.1	6.31	4.76
propyl butyrate	-	2.1	2.07	-	-	-	-	-	-	-	-	3.77	-	4.34	-	-	-	-
ethylidene diacetate	4.32	-	-	-	-	-	-	-	9.34	-	-	-	-	-	-	-	6.53	1.07
methyl pyruvate	48.64	-	2.23	0.23	1.51	-	-	-	0.2	0.41	-	-	-	120.75	-	-	-	0.26
ethyl caproate	49.03	-	45.34	98.78	20.07	82.93	-	-	76.59	106.93	47.6	-	71.63	42.92	50.3	85.29	73.89	35.62
isoamyl butyrate	-	-	-	-	-	-	-	-	4.05	-	11.94	-	-	-	-	-	-	-
n-hexyl acetate	96.55	213.8	221.37	77.05	41.65	87.46	74.8	75.54	113.87	70.97	106	104.64	64.27	70.54	52.86	67.06	73.8	44.78
isocyanic acid	-	-	-	-	-	-	-	-	120.1	-	-	-	-	-	-	-	-	-
isoamyl 2-methylbutyrate	-	82.34	10.78	28.26	-	217.03	-	-	-	58.1	146.33	28.15	131.52	52.18	26.41	-	-	35.1
isoamyl isovalerate	-	30.83	8.94	22.14	39.73	34.06	21.38	12.84	4.75	21.62	47.35	18.57	25.73	13.08	15.59	13.75	4.74	15.23
methyl lactate	-	17.5	-	-	-	22.97	25.07	-	45.42	-	17.08	-	26.73	13.93	18.66	-	-	21.24
butanoic acid, 2-methyl-, pentyl ester	-	12.58	-	5.6	-	53.82	0.9	-	-	3.97	38.28	2.84	22.63	10.65	1.23	-	-	3.97
ethyl lactate	0.63	-	-	0.34	3.14	0.73	0.97	0.75	1.19	0.55	0.55	0.62	0.72	0.44	0.63	0.63	0.36	-
hexyl isobutyrate	-	-	-	-	-	22.05	-	-	-	-	18.24	-	11.12	-	-	-	-	-

	CBR	CSW	XHR	XG	XHS	CM	CHN	CXZ	CGR	CYS	XM	CN	CGM	CSS	CD	CX	CHX	CG
butanoic acid, 3-methyl-, pentyl ester	-	7.52	-	24.95	-	13.94	-	-	-	12.71	16.71	2.75	7.26	3.33	3.67	-	-	1.89
hexyl formate	-	186.34	-	-	-	-	-	-	-	-	-	-	-	168.67	-	-	-	-
hexyl butyrate	-	-	-	-	-	-	-	-	-	26.86	-	-	-	69.34	5.6	-	-	-
heptyl isobutyrate	-	4.83	-	1.47	-	18.18	-	-	-	-	-	-	-	0.88	-	-	-	-
alpha-angelica lactone	21.63	5.17	-	4.39	2.1	-	-	2.13	24.23	2.69	-	3.93	40.93	15.22	15.85	2.97	3.65	4.71
hexyl 2-methylbutyrate	-	9.13	14.6	-	-	122.33	124.36	-	14.65	33.95	175.5	17.31	88.4	20.68	17.43	24.88	5.97	21.42
ethyl caprylate	2693.23	-	75.68	2376.54	-	3373.63	0.77	69.91	1872.78	0.86	2.42	-	-	1880.26	2696.15	-	-	1389.3
3-butenyl isothiocyanate	1.12	-	7.96	-	18.05	35.63	34.34	17.22	-	34.92	20.77	7.62	1.12	-	-	23.08	-	-
7-Methyloctanoic acid, methyl ester	0.35	-	1.37	-	-	0.4	13.45	-	-	13.4	7.43	1.12	-	-	-	-	-	-
hexyl isovalerate	-	118.3	-	-	-	49.4	-	-	-	-	49.99	32.24	15.03	-	-	-	-	-
n-hexyl valerate	-	-	-	10.81	-	49.47	-	-	-	17.24	-	32.2	65.86	-	-	-	-	-
ethylene glycol diacetate	-	-	11.51	7.19	204.21	-	-	-	-	-	-	-	-	-	-	-	-	-
3-hexenyl 2-methylbutyrate, (3z)-	19.4	-	116.91	-	-	-	5.92	-	-	-	177.47	39.76	-	-	-	-	-	12.88
3-hexenyl propionate, (3z)-	-	186.66	-	-	-	-	4.78	-	-	-	-	-	103.86	-	-	0.79	-	-
hexenyl valerate, (3z)-	2.35	11.13	2.63	4.7	-	-	3.46	-	-	5.96	26.27	4.54	6.49	1.47	-	-	-	3.74
3-hexenyl isovalerate, (3z)-	-	-	-	5.08	-	16.2	-	-	-	6.95	28.39	-	6.51	-	-	-	-	-
3,7-dimethyl-1,6-octadien-3-yl formate	-	-	-	-	-	-	-	-	-	139.33	-	-	-	-	-	-	17.43	32.15
linalyl acetate	101.15	954.51	-	-	-	421.62	1247.44	68.01	35.14	130.68	-	119.08	64.87	56.58	125.57	243.56	-	28.78
hexyl hexanoate	5.24	18.98	-	0.26	-	126.16	-	-	-	-	121.84	13.47	49.09	114.03	-	-	-	14.1
delta-valerolactone	-	-	5.6	-	-	-	-	-	-	-	16.3	13.59	20.1	11.66	9.48	6.66	-	-
butyrolactone	123.75	-	-	321.46	57.72	184.74	81.58	-	-	-	49.86	127.19	-	-	-	68.32	80.34	135.18
ethylene glycol, monoacetate	-	-	-	-	-	-	-	54.05	76.72	52.63	-	57.84	66.31	-	-	35.16	25.74	-
benzoyl isothiocyanate	-	-	-	-	-	-	-	-	-	-	155.54	-	-	-	-	-	-	-
vinyl hexanoate	-	-	4.78	-	20.55	1.26	-	0.65	3.32	-	0.44	-	-	5.72	-	-	0.55	-

	CBR	CSW	XHR	XG	XHS	CM	CHN	CXZ	CGR	CYS	XM	CN	CGM	CSS	CD	CX	CHX	CG
ethyl benzoate	2.05	1.75	-	-	1.28	0.8	2.68	0.84	1.58	1.92	0.39	1.41	0.73	1.01	2	0.79	0.52	1.14
cyclohexane, isothiocyanato-	-	14.7	-	0.94	2.65	-	-	2.24	-	-	-	5.71	-	7.21	-	-	1.1	-
4-hexanolide	3.01	-	2.28	10.11	5.01	1.16	-	1.02	1.42	2.26	-	1.19	-	0.83	1.43	1.43	1.61	0.97
alpha-terpinyl acetate	-	29.82	-	-	-	-	157.45	-	-	-	-	-	11.32	-	7.32	10.37	-	-
isobornyl acrylate	-	1.08	0.83	-	1.65	-	-	-	-	-	-	-	-	-	-	-	-	-
neryl isobutyrate	-	30.45	-	-	-	-	-	-	-	-	-	-	-	-	-	-	-	-
methyl 3-furoate	-	-	3.25	8.16E-06	-	-	-	-	-	-	-	-	-	-	-	-	-	-
methyl phenylacetate	-	-	-	-	-	-	-	0.55	0.52	1.19	-	-	-	-	-	-	-	-
geraniol acetate	2.5	38.06	-	-	-	13.33	36.72	-	-	-	-	-	-	-	-	9.11	-	-
4-hexen-1-ol, 5-methyl-2-(1-methylethenyl)-, 1-acetate	-	-	-	-	-	14.26	-	1.73	-	-	-	-	-	1.65	-	-	-	-
tetrahydro-4-methyl-2h-pyran-2-one	1.26	3.29	0.41	2.95	-	0.14	0.32	3.21	2.09	1.14	1.55	1.94	0.71	1.7	0.53	1.4	1.73	3.61
methyl salicylate	6.59	22.73	13.56	7.9	4.14	78.31	42.8	14.37	13.38	53.26	30.14	27.4	25.4	25.21	22.63	9.21	5.7	41.3
delta-hexalactone	2.9	3.17	1.89	6.32	11.56	-	2.94	1.27	3.01	4.01	0.84	2	1.48	1.46	2.31	3.69	0.69	0.63
acetic acid, 2-phenylethyl ester	0.83	1.25	-	-	-	5.85	-	-	2.27	1.39	0.8	1.38	2.1	3.64	2.31	0.65	-	2.42
isoamyl decanoate	-	18.76	3.64	2.95	-	-	2.26	-	0.52	3.83	-	-	5.4	4.08	4.49	4.43	2.39	2.14
2-naphthyl caprylate	-	8.67	-	-	-	-	-	-	-	-	-	-	-	-	-	-	-	-
isobutyraldehyde trimer	-	2.18	-	-	-	0.56	-	-	-	-	-	-	0.44	-	-	-	-	6.04
2,2,4-trimethyl-1,3-pentanediol diisobutyrate	-	-	-	-	-	0.32	3.22	-	-	-	-	-	-	0.45	0.61	-	-	2.36
tri-isobutylphosphate	51.11	62.28	51.78	57.98	44.11	42.51	140.92	24.45	29.4	69.97	28.32	54.56	32.95	26.54	20.88	40.66	32.77	35.03
3-hexenyl butyrate, (3z)-	4.47	-	2.29	-	-	-	-	-	-	-	-	-	-	-	-	-	-	-
pantolactone, (s)-	-	-	-	-	-	-	-	-	-	-	-	-	-	31.55	-	-	14.06	-
pantolactone	48.33	17.89	9.55	19.99	5.85	24.87	15.86	14.76	36.86	30.45	2.71	15.68	24.77	27.15	25.02	18.84	14.66	17.93

	CBR	CSW	XHR	XG	XHS	CM	CHN	CXZ	CGR	CYS	XM	CN	CGM	CSS	CD	CX	CHX	CG
methyl trans-cinnamate	-	-	-	-	-	2.07	-	-	-	-	0.46	-	-	3.5	-	-	-	-
bis(tert-butoxycarbonyl)oxide	-	-	-	-	-	-	-	-	-	-	-	-	-	-	0.98	-	-	-
ethyl cinnamate	5.41	7.44	3.64	6.49	2.17	8.86	5.36	1.36	4.32	2.5	0.45	9.14	3.18	1.3	1.55	0.66	-	-
2-hydroxy-gamma-butyrolactone	13.43	11.65	25.55	12	16.46	4.4	11.57	11.93	11.62	10.13	11.1	11.44	5.77	6.39	9.6	8.39	14.15	7.35
pentadecyl acetate	-	4.01	-	-	-	2.75	-	-	0.65	2.36	-	4.14	-	0.62	-	-	-	1.06
undecyl acetate	-	3.71	-	-	-	-	-	-	-	-	-	-	-	-	-	-	-	-
methyl palmitate	0.88	-	0.26	1.1	0.62	1	1.72	1.02	-	1.63	0.41	-	-	-	-	-	-	-
ethyl palmitate	-	-	2.16	2.39	1.31	2.17	16.44	3.46	2.82	2.46	-	3.86	3.52	-	-	-	-	-
oxacyclohexadecan-2-one	3	2.03	1.34	3.53	-	2.16	-	-	-	4.29	1.53	2.61	1.96	0.93	-	-	-	0.71
glyceryl 1-acetate	-	-	-	-	-	-	6	-	-	4.61	-	4.32	-	-	-	-	-	-
dimethyl phthalate	-	1.54	-	-	-	2.47	1.49	0.44	0.27	-	0.23	-	0.27	-	2.17	0.77	0.34	2.57
dihydroactinidiolide	58.4	9.24	24.24	90.34	81.33	30.44	51.62	48.67	32.83	25.71	1.38	19.84	16.75	17.03	10.82	35.61	15.42	11.24
mevalonolactone, (+/-)-	-	-	-	2.12	-	-	-	-	-	-	-	-	-	-	-	-	-	-
diisobutyl phthalate	1.23	1.77	1.73	3.06	1.07	1.58	2.85	1.64	1.39	2.16	1.62	1.71	1.01	1.09	0.9	1.02	1.08	2.36
3,4-dihydroxybutanoic acid gamma-lactone	3.19	1.89	1.62	-	0.72	1.23	-	1.24	2.51	2.08	0.93	1.28	1.51	1.77	3.19	-	-	1.81
dibutyl phthalate	1.76	1.17	0.91	3.18	1.13	0.9	1.78	1.09	0.92	3.52	0.55	-	0.56	0.76	1.64	0.82	0.73	-
Acids（18）																		
acetic acid	2762.29	4171.57	1317.71	4032.87	1557.48	3359.34	1441.57	1220.62	4587.35	1284.1	770.78	2485.05	3246.34	4072.65	2715.94	2384.14	2233.1	2894.34
oxalic acid	-	-	-	-	6.12	293.05	-	-	162.53	-	-	-	-	139.62	27.07	-	-	100.43
propionic acid	104.42	168.4	163.02	226.02	446.59	348	79.83	101.92	126.92	131.15	69.24	147.96	168.92	140.26	100.79	108.32	95.18	112.91
isobutyric acid	50.36	107.25	88.89	604.57	107.84	-	143.91	25.77	117.13	54.01	46.69	109.75	120.21	-	115.98	128.31	80.92	73.66
4-methyl-3-pentenoic acid	-	92.35	-	-	-	85.52	95.62	-	-	167.08	18.02	251.38	98.61	81.75	125.07	35.67	37.4	63.16
n-butyric acid	9.85	0.65	11.83	19.69	10.29	49.53	30.91	9.21	8.66	13.64	-	-	9.01	7.78	7.79	5.77	8.26	12.17
acrylic acid	-	-	-	-	-	-	-	-	0.72	-	-	-	-	3.12	10.05	-	-	0.53

	CBR	CSW	XHR	XG	XHS	CM	CHN	CXZ	CGR	CYS	XM	CN	CGM	CSS	CD	CX	CHX	CG
isovaleric acid	-	-	-	776.73	-	154.62	341.72	211.6	875.78	-	-	563.27	-	-	852.82	407.61	387.9	578.27
2-methylbutanoic acid	371.39	639.12	227.63	786.1	232.77	828.75	335.79	215.16	876.56	771.36	392.21	561.99	794.83	664.5	855.03	407.65	386.78	578.53
n-pentanoic acid	25.51	-	31.55	64.28	14.64	96.69	32.82	15.76	40.99	25.95	14.89	40.94	59.51	76.21	-	27.35	17.48	40.21
2-methylvaleric acid	0.35	-	-	-	-	2.3	0.43	0.72	0.67	-	-	3.82	0.29	0.95	3.33	0.21	0.32	1.49
isocaproic acid	19.36	68.05	13.91	85.61	2.94	245.91	27.35	8.49	32.19	118.08	41.16	86.39	116.67	126.9	61.33	7.77	13.49	26.63
angelic acid	-	-	110.65	33.5	-	31.36	-	-	98.92	3.05	2.24	6.42	7.13	-	15.75	12.3	12.18	-
hexanoic acid	129.07	47.62	126.51	264.69	155.18	166.18	64.54	88.77	117.48	33.97	35.85	59.66	104.78	265.84	100.97	126.56	85.31	58.46
2-ethylhexanoic acid	6.25	15.03	6.59	-	4.43	89.63	21.62	-	-	4.69	4.44	3.12	5	76.02	73.28	9.12	10.14	14.48
7-methyloctanoic acid	-	-	-	-	-	-	-	-	-	-	-	9.2	5.41	-	-	-	-	-
nonanoic acid	-	0.87	-	-	-	0.86	-	-	2.95	-	-	1.56	0.64	-	-	-	-	1.89
6-nonenoic acid, 8-methyl-, (e)-	-	-	-	-	-	-	-	-	-	-	-	8.77	-	-	-	-	-	-
Others（23）																		
2-methylpyrazine	0.24	8.19	32.55	11.42	8.11	-	-	-	-	6.68	6.83	-	-	-	-	-	-	-
2,5-dimethylpyrazine	33.98	30.36	53.07	67.72	40.19	30.98	100.98	31.92	13.12	47.26	41.45	26.85	-	-	25.16	51.07	66.77	-
2,3-dimethylpyrazine	0.47	1.07	33.15	66.52	2.42	8.05	6.95	-	-	8.55	4.75	-	-	0.52	0.54	0.19	0.56	4.15
2-ethyl-6-methylpyrazine	-	1.27	4.38	2	2.12	0.77	-	0.39	-	3.24	-	2.38	0.54	-	0.59	-	1.33	1.22
2,3,5-trimethylpyrazine	12.41	9.83	31.38	103.62	11.47	6.22	7.65	5.82	21.93	-	-	5.81	5.3	0.67	-	-	7.28	1.06
5-ethyl-2,3-dimethylpyrazine	-	0.93	-	15.22	-	-	-	-	-	-	-	-	-	-	-	-	-	-
2-isobutyl-3-methoxypyrazine	59.61	612.04	185.44	-	-	168.94	1.074948	24.61	25.55	488.25	579.72	406.25	396.81	83.58	76.1	30.93	25.77	5.56
diethyl ether	1327.02	1009.83	1095.11	1068.53	1.51.55	2177.22	3259.03	1228.39	1710.54	3287.09	1702.7	2348.55	2062.41	1628.94	1893.83	1965.88	1652.77	1011.35
dibutyl ether	-	4.05	0.21	-	-	13.41	7.67	9.06	9.53	10.08	16.46	8.28	10.46	6.1	7.32	-	9.88	3.13
dimethyl disulfide	-	26.45	0.56	5.21	5.21	7.75	18.41	2.19	4.18	8.34	6.68	7.39	4.15	8.27	7.56	5.35	3.18	5.6
1-methoxy-2-hydroxypropane	7.5	11.49	25.8	30.4	32.55	19.45	4.78	32.03	8.78	15.75	31.33	18.87	13.06	10.97	13.96	10.63	25.33	19.91
methyl propyl disulfide	1.58	1.42	1.77	2.4	1.72	1.84	2.12	1.46	1.58	2.14	2.61	2.15	2.32	1.69	2.23	2.66	1.56	1.22
allyl methyl disulfide	45.99	0.24	-	-	-	0.25	3.1	-	1.05	1.35	0.62	-	2.05	-	1.12	-	-	-

	CBR	CSW	XHR	XG	XHS	CM	CHN	CXZ	CGR	CYS	XM	CN	CGM	CSS	CD	CX	CHX	CG
butoxypropanol	1.28	-	-	-	-	-	-	-	-	-	2.89	-	-	-	-	0.71	-	-
ethylene glycol mono-n-butyl ether	4.55	-	0.15	-	-	4.14	-	13.4	17.22	22.11	3.57	6.88	2.5	-	9.75	25.13	-	1.7
methyl propyl ether	-	-	-	-	-	-	-	-	-	-	5.88	-	-	-	-	-	-	-
diallyl disulfide	30.39	149.01	33.88	26.35	19.16	-	159.96	13.23	40.82	53.21	307.16	-	-	-	29.74	18.82	6.74	23.86
diethylene glycol monomethyl ether	-	23.04	-	-	-	-	-	-	-	-	-	-	-	-	-	-	-	-
2-methoxyethanol	15.24	-	1.21	1.05	-	-	-	49.65	-	-	-	-	-	17.78	-	-	-	-
1,3-dimethoxybenzene	5.62	-	2.53	11.79	8.69	3.45	-	-	-	-	-	-	-	-	-	-	-	-
diethylene glycol mono-n-butyl ether	7.2	25.03	2.74	5.12	0.34	48.77	8.09	2.8	9.46	4.77	1.69	5.37	7.68	63.98	-	3.53	3.05	9.29
2,4,6-trichloroanisole	-	18.12	-	-	-	38.86	-	-	-	-	-	19.88	-	-	-	-	3.29	6.15
2,2-oxydipropanol	1.58	1.72	-	-	-	9.04	-	-	-	2.35	1.41	-	-	1.42	-	-	-	-

"-" indicates that it is not detected.
